# AI meets informed consent: a new era for clinical trial communication

**DOI:** 10.1093/jncics/pkaf028

**Published:** 2025-03-18

**Authors:** Michael Waters

**Affiliations:** Department of Radiation Oncology, Washington University in St Louis School of Medicine, St Louis, MO, United States

## Abstract

Clinical trials are fundamental to evidence-based medicine, providing patients with access to novel therapeutics and advancing scientific knowledge. However, patient comprehension of trial information remains a critical challenge, as registries like ClinicalTrials.gov often present complex medical jargon that is difficult for the general public to understand. While initiatives such as plain-language summaries and multimedia interventions have attempted to improve accessibility, scalable and personalized solutions remain elusive. This study explores the potential of Large Language Models (LLMs), specifically GPT-4, to enhance patient education regarding cancer clinical trials. By leveraging informed consent forms from ClinicalTrials.gov, the researchers evaluated 2 artificial intelligence (AI)-driven approaches—direct summarization and sequential summarization—to generate patient-friendly summaries. Additionally, the study assessed the capability of LLMs to create multiple-choice question-answer pairs (MCQAs) to gauge patient understanding. Findings demonstrate that AI-generated summaries significantly improved readability, with sequential summarization yielding higher accuracy and completeness. MCQAs showed high concordance with human-annotated responses, and over 80% of surveyed participants reported enhanced understanding of the author’s in-house BROADBAND trial. While LLMs hold promise in transforming patient engagement through improved accessibility of clinical trial information, concerns regarding AI hallucinations, accuracy, and ethical considerations remain. Future research should focus on refining AI-driven workflows, integrating patient feedback, and ensuring regulatory oversight. Addressing these challenges could enable LLMs to play a pivotal role in bridging gaps in clinical trial communication, ultimately improving patient comprehension and participation.

Clinical trials form a cornerstone of evidence-based medicine, offering patients access to novel therapeutics while serving an integral role in the advancement of scientific knowledge. Despite their crucial role, widely accessible materials to educate patients and the public about specific trial options remain lacking—a challenge that has been recognized for decades.^1^ Currently, trial registries like ClinicalTrials.gov serve as the primary resource for doctors and patients to explore available trial options. However, despite being publicly accessible, these platforms often use highly technical language that remains challenging for the general audience to understand.

Patients with cancer greatly benefit from access to clinical trial information but are particularly susceptible to misunderstandings. The volume of details, combined with the burden of their disease, can be overwhelming and distressful. While initiatives such as plain-language summaries and multimedia interventions have sought to improve comprehension,^2^ a scalable and personalized solution remains to be realized. Given this, the growing adoption of generative artificial intelligence (AI) in health care presents an opportunity to bridge this communication gap.

Large Language Models (LLMs), a pervasive subtype of generative AI text models, have shown remarkable promise across diverse applications in medicine, from summarizing medical literature to performing tasks that assist physicians in clinical decision-making.^3-5^ Given their ability to process and generate human-like text, the authors of *The use of large language models to enhance cancer clinical trial educational materials*^6^ present LLMs as an opportunity to revolutionize patient education when interacting with clinical trials by simplifying complex medical jargon from clinical trial registries into more accessible formats. The integration of AI-powered text generation into clinical trial education represents a novel and exciting direction for improving patient understanding and engagement. It also marks a novel use of LLMs in the clinical trial space above and beyond simple patient-trial matching.

Despite this potential, the adoption of LLMs in patient-facing medical roles has generated controversy. One major concern is their tendency to generate “hallucinations”—plausible yet incorrect or misleading statements.^4^ The reliability of LLM-generated content in high-stakes domains such as oncology remains a pressing question. Additionally, ethical concerns regarding bias, transparency, and the regulatory oversight of AI-driven patient education warrant further discussion.^4^ Ensuring the accuracy, fairness, and interpretability of AI-generated medical content is discussed, in detail, in this manuscript.

To evaluate LLM potential in this space, this study examines the capabilities of LLMs, specifically GPT-4, to enhance educational materials for cancer clinical trials. Namely, the study utilized informed consent forms (ICFs) from ClinicalTrials.gov to generate patient-friendly summaries and multiple-choice question-answer pairs (MCQAs) pertaining to several oncologic clinical trials. Informed consent forms are official documents required for clinical trial launch and enrollment that explain the purpose, risks, benefits, and procedures of a clinical trial, typically written in technical language that can be difficult for patients to understand. The researchers aimed to transform these complex documents into simpler, more digestible summaries that could help patients make informed decisions about participation in clinical trials.

With this goal in mind, the authors utilized 2 approaches to generate ICF summaries. The first method, direct summarization, involved asking the LLM to generate a concise summary of an ICF in 1 step. The second method, sequential summarization, involved a multi-step process to systematically refine the summary for improved clarity and accuracy. Namely, GPT-4 was first prompted to extract relevant sections from the ICFs, identifying key details such as study objectives, procedures/alternative procedures, risks, costs, and potential benefits. In the sequential workflow, the extracted content was processed in stages, with each step focusing on restructuring and simplifying complex medical language while preserving essential information. Additionally, the study explored how well LLMs could create MCQAs to assess a patient’s understanding of the summarized content.

The authors demonstrate the novel finding that both direct and sequential summarization approaches produced patient-friendly summaries that were significantly easier to understand than the original ICFs. Additionally, sequential summarization led to summaries with slightly fewer inaccuracies or missing details. The MCQAs generated by LLMs demonstrated high agreement with crowdsourced human annotators. Notably, greater than 80% of surveys indicated that the summary improved understanding of a particular clinical trial evaluated in the work: BROADBAND. These findings underscore the potential of LLMs to transform how clinical trial information is communicated, making complex medical documents more accessible and patient-friendly. This aligns with broader efforts to integrate AI-driven solutions into health-care education, addressing longstanding challenges in patient comprehension and engagement.

The integration of AI, particularly LLMs, into patient education is an evolving field. While AI has been extensively explored for clinical decision support,^5^ medical text summarization,^3^ and chatbot-assisted patient interactions,^4^ their role in enhancing awareness and understanding of clinical trials is unexplored. Preliminary efforts have evaluated LLMs for patient-trial matching.^7^^,^^8^ However, beyond trial matching, LLMs have not been widely utilized for other aspects of clinical trials engagement, such as enhancing patient education, generating informed consent summaries, or improving public outreach.

Previous studies have consistently shown that complexity of clinical trial documents poses a significant barrier to patient comprehension and recruitment. Strikingly, the readability of clinical trial documents has consistently been shown to exceed the recommended comprehension guidelines established across multiple disciplines and endorsed by professional organizations, including the American Medical Association (AMA).^9-11^ This complexity can lead to low recruitment rates and disparities in trial participation.^12^ As an illustrative example, a nationwide study by Perni et al. assessed the readability and specificity of ICFs for patients undergoing radiotherapy.^13^ Their findings revealed that while all academic radiotherapy departments required written informed consent, the vast majority of these forms did not meet the recommended readability levels. Using 7 validated readability indices, they found that the mean readability of consent forms ranged from grade level 10.6 to 14.2, far exceeding the recommended sixth- to eighth-grade level. Notably, only 8% of forms met the eighth-grade threshold.

As well known as the problem is, little exists in the published literature to address this challenge. Kumar et al. previously explored the use of GPT-4 to generate clinical note summaries for patient audiences, highlighting the model’s ability to reformat structured data into narrative descriptions; however, this functionality has not been extended to clinical trials.^14^ The present study builds on this work by assessing GPT-4’s ability to create patient-focused summaries and educational materials, marking a meaningful shift in AI applications from research-facing to patient-centered tools. The workflow presented in this manuscript has the potential to address previously described challenges, such as the technical jargon found in cancer trial websites,^15^ language barriers in maternal health trials,^16^ and the excessive complexity of patient information sheets in neuro-oncology trials.^17^

This study highlights the potential of LLMs to improve clinical trial education by generating patient-friendly materials that enhance comprehension and accessibility. The ability of LLMs to transform complex medical jargon into digestible summaries represents a promising advancement in patient engagement. However, challenges such as AI hallucinations, content accuracy, and regulatory oversight must be addressed before widespread adoption. Given this, the authors point out that even manually written patient facing recruitment and consent materials standardly undergo additional human review by institutional review boards. Future research should focus on refining AI-driven workflows, integrating real-world patient feedback, assessing and tabulating LLM performance across disparate types of clinical trials, and ensuring robust validation through large-scale clinical evaluations. Additionally, exploring personalization strategies that tailor educational materials to individual patient needs could further enhance the impact of AI-generated content. By addressing these challenges, LLMs have the potential to bridge critical gaps in clinical trial communication, ultimately improving patient understanding and participation.

## Data Availability

No new data were generated or analyzed for this editorial.
